# Bright blue-shifted fluorescent proteins with Cys in the GAF domain engineered from bacterial phytochromes: fluorescence mechanisms and excited-state dynamics

**DOI:** 10.1038/srep37362

**Published:** 2016-11-18

**Authors:** Yusaku Hontani, Daria M. Shcherbakova, Mikhail Baloban, Jingyi Zhu, Vladislav V. Verkhusha, John T. M. Kennis

**Affiliations:** 1 Department of Physics and Astronomy, VU University Amsterdam, Amsterdam 1081 HV, The Netherlands; 2 Department of Anatomy and Structural Biology and Gruss-Lipper Biophotonics Center, Albert Einstein College of Medicine, Bronx, New York 10461, USA; 3 Department of Biochemistry and Developmental Biology, Faculty of Medicine, University of Helsinki, Helsinki 00290, Finland

## Abstract

Near-infrared fluorescent proteins (NIR FPs) engineered from bacterial phytochromes (BphPs) are of great interest for *in vivo* imaging. They utilize biliverdin (BV) as a chromophore, which is a heme degradation product, and therefore they are straightforward to use in mammalian tissues. Here, we report on fluorescence properties of NIR FPs with key alterations in their BV binding sites. BphP1-FP, iRFP670 and iRFP682 have Cys residues in both PAS and GAF domains, rather than in the PAS domain alone as in wild-type BphPs. We found that NIR FP variants with Cys in the GAF or with Cys in both PAS and GAF show blue-shifted emission with long fluorescence lifetimes. In contrast, mutants with Cys in the PAS only or no Cys residues at all exhibit red-shifted emission with shorter lifetimes. Combining these results with previous biochemical and BphP1-FP structural data, we conclude that BV adducts bound to Cys in the GAF are the origin of bright blue-shifted fluorescence. We propose that the long fluorescence lifetime follows from (i) a sterically more constrained thioether linkage, leaving less mobility for ring A than in canonical BphPs, and (ii) that π-electron conjugation does not extend on ring A, making excited-state deactivation less sensitive to ring A mobility.

Near-infrared (NIR) fluorescent proteins (FPs) are of great interest for *in vivo* imaging of mammals. Because an optical transparency window of mammalian tissues exists in the NIR region, 650–900 nm, where water, hemoglobin and melanin absorptions are minimal^1^,^2^, noninvasive deep-tissue imaging can be achieved with NIR FPs. So far, many kinds of GFP-based FPs have been engineered and applied to real-time optical imaging in life sciences^3^. However, probably due to the limitation of the extent of the conjugated π-electron system, the absorption in engineered GFP-like proteins is limited to about 610 nm^4^,^5^ and fluorescence emission to about 675 nm^6^.

Phytochromes are red-light absorbing photoreceptors using bilin chromophores^7^,^8^,^9^. The first fluorescent phytochrome, derived from the cyanobacterial phytochrome Cph1 was reported by Fischer and Lagarias^10^. Subsequently, NIR FPs have been engineered from bacterial phytochromes (BphPs), derived from *Deinococcus radiodurans Dr*BphP^11^,^12^ and *Rhodopseudomonas palustris Rp*BphP^1^,^13^,^14^,^15^,^16^. BphPs bind a biliverdin (BV) tetrapyrrole compound, which is a heme degradation product. Therefore, BV is endogenously present in all mammalian tissues^11^,^17^, which allows to use BphP-derived NIR FPs as easily as GFP-like FPs, without adding chromophore exogenously. The photosensory core of phytochromes comprises three domains: PAS, GAF and PHY. The PAS-GAF fragment, which has a molecular weight of about 35 kDa in the monomeric form^16^, is adequate for chromophore binding. BphPs have a Cys residue in the PAS domain that covalently binds to BV via the C3^2^ atom in ring A^18^,^19^. In the dark, canonical BphPs adopt a 670–690 nm absorbing state known as the Pr state. Upon irradiation at these wavelengths, BphPs undergo photoisomerization in C15=C16^20^,^21^, resulting in a photoproduct Lumi-R^22^,^23^,^24^,^25^,^26^,^27^,^28^,^29^,^30^, which then thermally relaxes to a 740–760 nm absorbing state termed Pfr^31^.

In recent years, several blue-shifted bright NIR FPs have been engineered from wild-type *Rp*BphP1, *Rp*BphP2, and *Rp*BphP6^15^, such as BphP1-FP (the brightest FP among current BphP-derived NIR FPs)^16^, iRFP682^14^ and iRFP670^14^, respectively. In these NIR FPs, the PHY domain was truncated, and photoisomerization was blocked through mutation of key residues in the GAF domain. Furthermore, a Cys residue was introduced in the GAF domain: Cys253 in BphP1-FP, Cys244 in iRFP670 and Cys249 in iRFP682, enabling BV to covalently bind to cysteine residues in the PAS and GAF domains^14^,^15^. These Cys residues in the GAF domain of BphP1-FP, iRFP670 and iRFP682 were introduced at the same position as the conserved Cys residues in plant and cyanobacterial phytochromes, which bind phycocyanobilin (PCB) or phytochromobilin (Pϕ, capical B)^7^ (Fig. S1). The blue-shifted NIR FPs have been successfully used in deep-tissue imaging and, moreover, enabled multicolor *in vivo* imaging^14^,^32^,^33^.

More recently, a crystal structure of BphP1-FP/C20S variant has been determined^16^. BV binds to Cys253 in the GAF domain in two ways, either via the C3^1^ atom or via the C3^2^ atom in an approximate 1:1 stoichiometry (Fig. 1). The unique chromophore species that result from this binding appear to be responsible for the spectral blue shift and the high fluorescence quantum yield. Yet, real-time studies of the picosecond dynamics on the excited states are required to understand molecular mechanisms of fluorescence more deeply.

Here we report the ultrafast photodynamics in BphP1-FP, iRFP670 and iRFP682 with femtosecond transient absorption and picosecond fluorescence spectroscopy. Furthermore, we analyze the ultrafast dynamics in their mutants that have a Cys residue in the GAF or PAS domains, or no Cys at all. We aim to gain insights into the molecular origin of the blue-shifted and bright BphP1-FP fluorescence, combining ultrafast spectroscopic results with previous biochemical and structural analyses. Elucidation of the molecular mechanisms of fluorescence emission should give insight into rational design of enhanced NIR-FPs with higher quantum yield and various absorption and emission wavelengths.

## Methods

### Protein expression and purification

The BphP1-FP, iRFP670 and iRFP682 genes were cloned into the pBAD/His-B vector (Invitrogen). Site-specific mutagenesis was performed using QuikChange kit (Stratagene). The proteins with polyhistidine tags on the N-termini were expressed in LMG194 bacterial cells (Invitrogen) bearing the pWA23h plasmid encoding heme oxygenase under the rhamnose promoter^14^. To initiate protein expression, bacterial cells were grown in RM medium supplemented with ampicillin, kanamycin and 0.02% rhamnose for 5 h at 37 °C. Then 0.002% arabinose was added and bacterial culture was incubated for additional 12 h at 37 °C followed by 24 h at 18 °C. Proteins were purified using Ni-NTA agarose (Qiagen). Ni-NTA elution buffer contained no imidazole and 100 mM EDTA. The elution buffer was substituted with PBS buffer using PD-10 desalting columns (GE Healthcare). For spectroscopic experiments, the samples were diluted in PBS buffer composed of 137 mM NaCl, 2.7 mM KCl, 10 mM Na_2_HPO_4_ and 2 mM KH_2_PO_4_ at pH 7.4.

### Steady state absorption and fluorescence spectroscopy

Steady-state absorption spectra were recorded using a UV/Vis spectrometer (Cary 4000, Agilent) at room temperature (~20 °C). The samples were diluted with PBS buffer (pH 7.4) and measured in a 1 mm pathlength quartz cuvette (100-QS, Hellma Analytics). Steady-state fluorescence spectra were measured with a Vis/Near-IR spectrometer (FluoroMax, Horiba) at the room temperature (~20 °C). The samples were diluted to an absorbance of 0.1 per cm to prevent reabsorption and measured in a 10 mm pathlength PMMA cuvette (759150, Brand). The excitation wavelength was 600 nm for the fluorescence measurements.

### Femtosecond transient absorption spectroscopy

Femtosecond transient absorption measurements were performed with a femtosecond pump-probe setup described previously^34^,^35^ at room temperature (~20 °C). A seed pulse from a Ti:Sapphire oscillator (Vittesse, Coherent; 800 nm, 82 MHz, 50 fs) was amplified to 4.5 W by using a Ti:Sapphire regenerative amplifier (Libra, Coherent; 800 nm, 1 kHz, 35 fs) with a Nd:YLF Q-switched high-power pump laser (Evolution, Coherent; 527 nm, 1 kHz). The beam was split into two paths as for pump and probe respectively. On the pump path, the beam was guided into an optical parametric amplifier (OPerA Solo, Coherent) to generate tunable pulses for excitation, and focused on the sample with a diameter of 150 μm. The pump pulse was progressively delayed with respect to the probe using a 60 cm long delay stage (IMS-6000, Newport) to cover a time window up to ~4 ns. The excitation wavelengths were selected as 590 nm, 630 nm, 640 nm or 660 nm depending on the sample. Narrow band interference filters (Δλ~10 nm) for the excitation wavelengths were inserted on the pump path. On the probe path, the beam was attenuated and focused on a 2 mm sapphire plate to generate white light for probing. A spectral range of 530–830 nm of the white light probe pulse was detected by a 256-segment photodiode array^36^. The polarization of pump and probe was set at the magic angle (54.7°). The samples in 1-mm cuvettes (100-QS, Hellma Analytics) with an absorbance of 1.5 per cm at the absorption maximum were set on a home-built vibrating sample holder in order to avoid damage to the samples.

### Picosecond time-resolved fluorescence spectroscopy

Time-resolved fluorescence measurements were carried out with a streak camera setup, as described previously^37^,^38^,^39^ at room temperature (~20 °C). Femtosecond laser pulses from an integrated Ti:Sapphire oscillator (Vitesse, Coherent, 800 nm, 80 MHz, 100 fs) was incorporated into a Ti:Sapphire regenerative amplifier (RegA, Coherent, 800 nm, 50 kHz, 100 fs). The beam was input to an optical parametric amplifier (OPA9400, Coherent) to create tunable excitation pulses. The excitation pulse was focused on a 3 mm pathlength quartz cuvette (102.251-QS, Hellma Analytics). The samples had an absorbance of 0.25 per cm at their absorbance maximum. The samples were excited at 590 nm, 630 nm, 640 nm or 660 nm. A narrowband interference filter (Δλ~10 nm) for respective excitation wavelength was located on the pump path. The applied excitation pulse energy was ~4 nJ, and the polarization between the excitation beam and emission was set at the magic angle (54.7°). Fluorescence from the sample was collected perpendicularly to the pump beam and focused into a slit using an achromatic lens and the wavelengths were resolved by a spectrograph (250IS, Chromex). Wavelength-resolved fluorescence was converted to electrons at the photocathode and a variable voltage was applied to sweep electrodes to collect the time-resolved fluorescence spectra on a streak camera (C5680, Hamamatsu).

### Data analysis

Global analysis was performed for the transient absorption spectra using the Glotaran program^35^,^40^. With global analysis, all wavelengths are analyzed simultaneously with a set of common time constants^41^. A kinetic model was applied consisting of sequentially interconverting, evolution-associated difference spectra (EADS), *i.e*. 1 → 2 → 3 → … in which the arrows indicate successive mono-exponential decays of a time constant, which can be regarded as the lifetime of each EADS^41^. The first EADS corresponds to the difference spectrum at time zero. The first EADS evolves into the second EADS with time constant *τ*_1_, which in turn evolves into the third EADS with time constant *τ*_2_, etc. The procedure clearly visualizes the evolution of the intermediate states of the protein^42^. Decay-associated difference spectra (DADS) indicate the spectral changes with parallel decay channels and independent decay time constants. It is important to note that parallel and sequential analysis are mathematically equivalent and yield identical time constants^39^. The transient fluorescent spectra were analyzed with parallel decaying components, which yield decay associated spectra (DAS)^41^. The standard errors in time constants were 2–5% the transient absorption data, and less than 1% for the transient fluorescence data in each decay component.

## Results

### Steady-state absorption and fluorescence spectroscopy

Steady-state absorption and fluorescence spectra of BphP1-FP and its C20S, C253I, C20S/C253I mutants are shown in Fig. 2a,b. The absorption of BphP1-FP and BphP1-FP/C20S peaks around 640 nm, while the other two mutants have maximum absorption around 670 nm. Fluorescence spectra of BphP1-FP and BphP1-FP/C20S have a maximum around 670 nm, while fluorescence of mutants BphP1-FP/C253I and BphP1-FP/C20S/C253I have a maximum around 700 nm. Compared to the wild-type *Rp*BphPs, which absorb at around 700 nm and fluoresce around 720 nm^19^,^39^,^43^,^44^,^45^, both of the absorptions and fluorescence of BphP1-FP and BphP1-FP/C20S has been strongly blue-shifted by about 60 nm. For mutants BphP1-FP/C253I and BphP1-FP/C20S/C253I, only moderate blue-shifts of absorption and fluorescence by ~20–30 nm were observed. In BphP1-FP and BphP1-FP/C20S, broader absorption was observed on the red edge compared to BphP1-FP/C253I and BphP1-FP/C20S/C253I. Similar observations were made in iRFP670 and iRFP682 and their corresponding mutants (Fig. S2). These results are consistent with previous work^14^,^16^,^46^.

### Time-resolved fluorescence spectroscopy

Picosecond time-resolved and spectrally resolved fluorescence spectroscopy was conducted to investigate the emission processes on BphP1-FPs, iRFP670 and iRFP682. Selected time-resolved emission spectra and decay associated spectra (DAS) on BphP1-FP are shown in Fig. 3. The excitation wavelength was 640 nm. Two exponential decay components were required for the fitting: *τ*_f2_ (1.56 ns) with a DAS peaking around 670 nm and *τ*_f1_ (0.35 ns) with its DAS peaking around 700 nm. The blue component *τ*_f2_ was dominant on the emission process, with amplitude of about 93% of the initially excited molecules.

Experiments on the BphP1-FP/C20S mutant gave very similar results to BphP1-FP, with a minor short-lifetime emission component *τ*_f1_ (0.34 ns) at around 700 nm, and a dominant long-lifetime component *τ*_f2_ (1.47 ns) at 670 nm (Fig. 4a). On the other hand, for the BphP1-FP/C253I and BphP1-FP/C20S/C253I mutants, no 670 nm emission was observed (Fig. 4b,c). In BphP1-FP/C253I, two emission components decaying with 0.26 ns (centered at 700 nm) and 0.88 ns (centered at 710 nm) were observed. Meanwhile, in BphP1-FP/C20S/C253I, two emission components centered at 700 nm decaying with 0.26 ns and 0.93 ns were observed.

Results for iRFP670 and iRFP682 are presented in Fig. 5, with global fitted time constants and DAS. Blue-shifted and long-lifetime components were observed only in iRFP670, iRFP670/C10A, iRFP682 and iRFP682/C15S. On the other hand, in the iRFP670/C244S, iRFP670/C10A/C244S, iRFP682/C15S and iRFP682/C15S/C249S mutants, only red-shifted and shorter-lifetime components were found, similar to the corresponding BphP1-FP mutants (Figs S3 and S4).

The time-resolved emission spectra shown in Figs 3 and 4 demonstrate that BphP1-FP, which has Cys253 in the GAF domain and Cys20 in the PAS domain, has a dominant fluorescence component (>90% amplitude) with a blue-shifted emission at 670 nm and long fluorescence lifetime of 1.56 ns. The experiments on the cysteine mutants clearly demonstrate that this blue-shifted, long-lived emission arises from BV bound to Cys253 in the GAF domain: upon deletion of Cys20 in the PAS domain, the emission wavelength and lifetime remains essentially the same. Upon deletion of Cys253 in the GAF domain, only shorter-lived emission components around 700–710 nm are observed. For iRFP670 and iRFP682 and their cysteine mutants, similar results were obtained although their fluorescence lifetimes were somewhat shorter than in BphP1-FP.

The BphP1-FP/C20S/C253I mutant shows weak fluorescence at 700 nm (decay with 0.26 ns and 0.93 ns, Fig. 4) even though it does not have a covalent bond with a cysteine according to the zinc staining^16^. This observation implies that BV has been incorporated non-covalently into the GAF domain pocket^18^, and has a red-shifted fluorescence component at 700 nm. The two fluorescence species of BphP1-FP/C253I at 710 nm and 700 nm are assigned to a BV covalently bound to Cys20 and a BV that non-covalently incorporated into the protein, respectively. In the corresponding Cys mutants of iRFP670 and iRFP682, the same characteristics were observed (Figs S3 and S4, ref. 46 for the zinc staining).

### Transient absorption spectroscopy

Femtosecond transient absorption spectroscopy was performed to characterize the primary photoreactions in BphP1-FP, iRFP670, iRFP682 and their mutants. Figure 6a shows raw transient absorption spectra in BphP1-FP at selected delay times with excitation at 640 nm. These data were globally fitted with three components, with lifetimes of *τ*_a1_ (7.6 ps), *τ*_a2_ (0.20 ns) and *τ*_a3_ (1.52 ns), and the resulting EADS and DADS are shown in Fig. 6b,c, respectively. For the BphP1-FP mutants, transient absorption spectra were fitted with three components as well (Fig. S5). The negative ΔA signal in Fig. 6b between 570–670 nm originates from ground state bleaching (GSB), and the positive ΔA signals around 530 nm and 700 nm indicate excited state absorption (ESA). As it can be seen from the EADS and DADS in Fig. 6c, the 1.52 ns component was clearly dominant and indicates loss of excited states, in agreement with the streak camera results. The 0.20 ns component denotes a minor excited-state decay component and corresponds to the minor 0.35 ns component from the streak camera experiments (Fig. 3b). In the early time range, transient absorption spectra only slightly changed with a time constant of 7.6 ps, which is interpreted as a relaxation process in the excited state^27^.

The global analysis indicates that no long-lived species are formed in the BphP1-FP (Fig. 6b,c) in contrast to wild-type *Rp*BphPs that form the photoproduct Lumi-R^27^,^39^. In agreement, time traces at selected wavelengths on transient absorption spectra of BphP1-FP are shown in Fig. 6d, clearly showing that the transient absorption signal almost completely decayed in 3 ns. These observations indicate that the BV C15=C16 photoisomerization that in native BphPs results in Lumi-R formation has been successfully impeded.

Similarly to BphP1-FP, three time constants were required to adequately describe the transient absorption data of iRFP670 and iRFP682, with 3.2 ps, 0.08 ns and 1.08 ns in iRFP670, and with 3.5 ps, 0.12 ns and 1.12 ns in iRFP682 (Fig. 7). Furthermore, three components were required to fit iRFP670/C10A, iRFP670/C10A/C244S, iRFP682/C15S, iRFP682/C249S as were the mutants of BphP1-FP (Figs S3 and S4). In iRFP670/C244S and iRFP682/C249S, two components were sufficient for global fitting with time constants of ~10 ps and ~0.6 ns.

The shape of the transient absorption spectra reported here merits some discussion. A pronounced ESA band near 700 nm that is red-shifted with respect to the ground state absorption is observed in BphP1-FP (Fig. 6) and iRFP670 (Fig. 7a) and their cysteine mutants, (Figs S3a,c,e and S5) and iRFP682 (Fig. 7b), while in the cysteine mutants of iRFP682 no or very little of such absorption is observed (Fig. S4a,c,e). The time evolution of the ΔA signal around 700 nm is indistinguishable from the ESA at 530 nm (Fig. S6), which solidifies the assignment of this signal to ESA. Almost all biliproteins show such a structured ESA at varying intensities, in phytochromes^23^,^27^,^28^,^39^,^47^ as well as biliprotein light harvesting antennae^48^. Such ESA may either be red-shifted, blue-shifted, or overlap with respect to the ground state absorption. Even among BphPs of the same species (*R. palustris*) and iRFPs derived thereof, the spectral position of the ESA has been shown to vary significantly^27^,^39^,^47^. In previous work it was demonstrated for *Rp*BphP2 and *Rp*BphP3 that the spectral position of this ESA is highly sensitive to the protein matrix, involving interactions between BV ring D with charged and hydrogen-bonding amino acid side chains:^27^ In the case of the iRFP682 cysteine mutants (Fig. S4a,c,e), the ESA either has a low amplitude or absorbs near the Pr absorption maximum, and is therefore entirely compensated by the ground state bleach and stimulated emission. Note that in plant phytochrome, such apparent absence of ESA was also observed^22^.

Because of the aforementioned variability of ESA in the transient absorption spectra, which compensates the ground state bleach and stimulated emission in an unpredictable manner, one cannot reliably associate the minima, maxima and zero crossings in the transient absorption spectra of Figs 6, 7, S3, S4 and S5 with the distinct spectral forms as observed in time-resolved fluorescence. For this reason, we make such assignments exclusively on the basis of the time-resolved fluorescence streak camera results. Given that the short-lived minor decay components in the transient absorption data have similar time constants as those derived from the streak camera, we conclude that they probably arise from the red-shifted population.

Furthermore, one may consider the possibility that the strong absorption near 700 nm could be due to a primary ground-state photoproduct, given that the canonical photoproduct Lumi-R (which has isomerized from the 15Za conformer to 15Ea) is expected to absorb near such wavelengths. We will show here that this is not the case. First, we note that the transient absorption and the streak camera experiments agree on the measured lifetimes, so we can assign the time constants in the transient absorption clearly to excited-state decay, and not ground-state product decay. Thus, if a transient product would be formed it would need to coexist with the singlet excited state during the fluorescence lifetime. With these conditions, the idea that the positive band arises from a primary photoproduct is highly unlikely, given that the 700 nm absorption rises within the instrument response of 100 fs. If it were a primary photoproduct, it would have to be formed in less than ~50 fs. It is well-established that in wild type phytochromes, the isomerized Lumi-R product rises on a much slower timescale of tens to hundreds of picoseconds from the Pr state^22^,^23^,^24^,^27^,^29^,^39^. Even if this were to happen, this putative product would then fortuitously need to have a lifetime that is exactly the same as the singlet excited state lifetime. We note that the biliprotein light harvesting antenna PC645 from cryptophyte algae shows a similar transient absorption signal red-shifted with respect to the ground state^48^, very similar to that of BphP1-FP. These type of light harvesting antennae have evolved for optimal light harvesting and show no transient photoproducts of their bilin chromophores whatsoever. Thus, we may safely conclude that the 700 nm transient absorption band corresponds to excited-state absorption, and not to a ground-state photoproduct.

### Kinetic isotope effects on the fluorescent lifetime

To investigate the fluorescence deactivation mechanisms in the presently studied NIR FPs, time-resolved fluorescence experiments in H_2_O and D_2_O solutions were conducted. Figure 8 shows DAS and time traces on BphP1-FP in H_2_O and D_2_O with 640 nm excitation. The DAS in H_2_O and D_2_O are almost identical, but the time constants are significantly different: 0.35 ns and 1.56 ns in H_2_O, and 0.48 ns and 2.89 ns in D_2_O. The H/D kinetic isotope effects (KIEs), defined as the fluorescence lifetime in D_2_O divided by the lifetime in H_2_O, on *τ*_a1_ and *τ*_a2_ on BphP1-FP were 1.4 and 1.9, respectively. For the BphP1-FP/C20S, C253I and C20S/C253I mutants, significant KIEs were observed as well (Table S1). Moreover, significant H/D KIEs were observed in iRFP670, iRFP682 and their mutants (Fig. S7 and Table S1). In the time region shorter than ~0.8 ns, a longer lifetime carries a larger KIE as in other iRFPs^47^ and wild-type BphPs^27^,^39^. On the other hand, a KIE of ~1.8 was observed for all lifetimes longer than ~0.8 ns (Fig. S8). The values of the large KIEs correspond to those of other red-shifted NIR FPs: iRFP702, iRFP713 and iRFP720^47^. The transient absorption data showed similar isotope effects (Table S1).

Significant KIEs were detected in fluorescence decays (Figs 8, S7 and S8) consistent with those of other iRFPs^47^, wild-type BphPs and their point mutants^27^,^39^, indicating that excited state proton transfer (ESPT) may constitute a significant non-radiative decay process, as discussed before^27^,^39^,^47^,^49^. Furthermore, KIEs in blue-shifted BphP1-FP (KIE: 1.9), iRFP670 (1.7) and iRFP682 (1.7) are as large as red-shifted iRFP702 (1.8), iRFP713 (1.8) and iRFP720 (1.9) while lifetimes of the blue-shifted NIR FPs are approximately twice larger^47^, which could imply that that the deactivation mechanism of the blue-shifted NIR FPs is similar to that of the red-shifted NIR FPs.

## Discussion

### Molecular basis for blue-shifted long-lived fluorescence in engineered bacterial phytochromes

**Figure 1:**
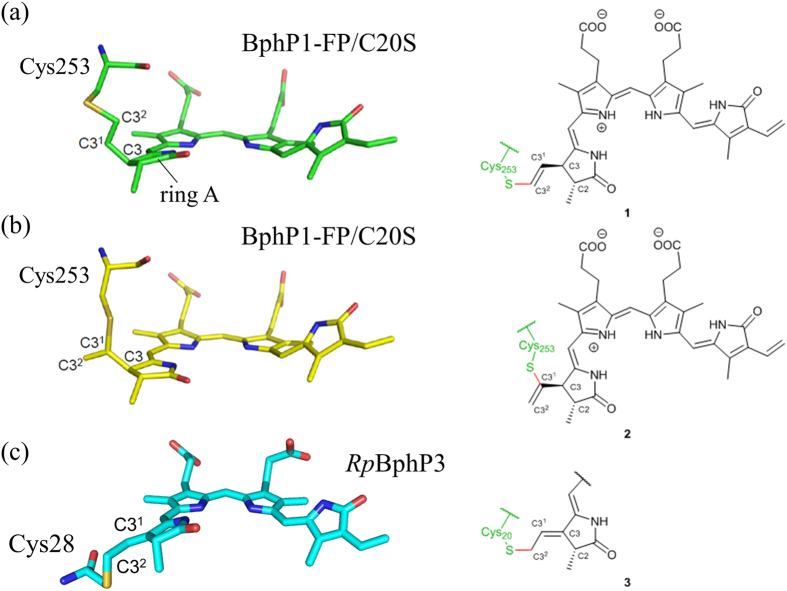
Structures of the BV adducts in engineered fluorescent bacterial phytochromes and in native bacterial phytochrome. (**a,b**) The engineered BphP1-FP/C20S (pdb code 4XTQ)^16^ and (**c**) native *Rp*BphP3 (*Rhodopseudomonas palustris*, pdb code 2OOL)^45^.

Our results have shown that the fluorescence of BphP1-FP is blue-shifted, bright and long-lived as compared to that of other native and engineered BphPs, which confers important advantages on this type of fluorescent proteins for *in vivo* multicolor imaging. We now discuss the molecular basis of these key properties. In previous work on BphP1-FP/C20S, its high-resolution X-ray structure was reported^16^. In that work, two distinct models of BV binding to Cys253 in the GAF domain were proposed, *i.e*. via the BV C3^2^ atom (**1**, Fig. 1a) and via the BV C3^1^ atom (**2**, Fig. 1b) in a ~1:1 stoichiometric ratio^16^. In contrast, the native BV adduct of the the similar *Rp*BphP3 protein (**3**, Fig. 1c) binds BV to Cys28 in the PAS domain. In both BV adducts of BphP1-FP/C20S, the C3 atom is sp^3^ hybridized resulting in an upward orientation of its substituent toward Cys253, and hence does not form the double bond with C3^1^ or C2 that is present in native bacterial phytochromes^19^,^43^,^45^. This molecular structure implies that the π-conjugated system of both BV adducts becomes shorter by one double bond as compared to BV bound to native bacterial phytochrome (cf. **1** and **2** *vs*. **3**, Fig. 1) and consequently, their absorption is 30–40 nm blue-shifted. In fact, the proposed conjugated π-electron system is identical to that of PCB or PФB bound to plant or cyanobacterial phytochromes^50^,^51^, and accordingly their absorption and emission maxima are nearly identical^10^,^22^. Recent work on the terminal phycobilisome emitter L_CM_ demonstrated a ZZZssa conformation for its PCB chromophore, like that in phytochrome, with its absorption and emission wavelength maxima very similar to that of BphP1-FP, plant and cyanobacterial phytochrome^52^. This indicates that the conjugated π-electron system of the chromophore constitutes the main determinant of absorption (~640–650 nm) and emission maxima (~670 nm), with a minor contribution from the specific protein environment.

**Figure 2:**
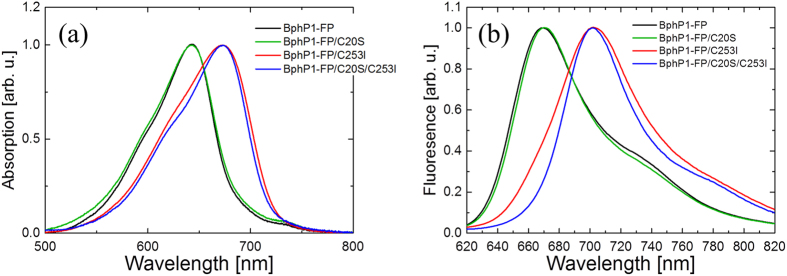
Steady-state absorption and fluorescence of BphP1-FP variants. (**a**) Absorption spectra and (**b**) fluorescence emission spectra of BphP1-FP and its C20S, C253I and C20S/C253I mutants are shown.

**Figure 3:**
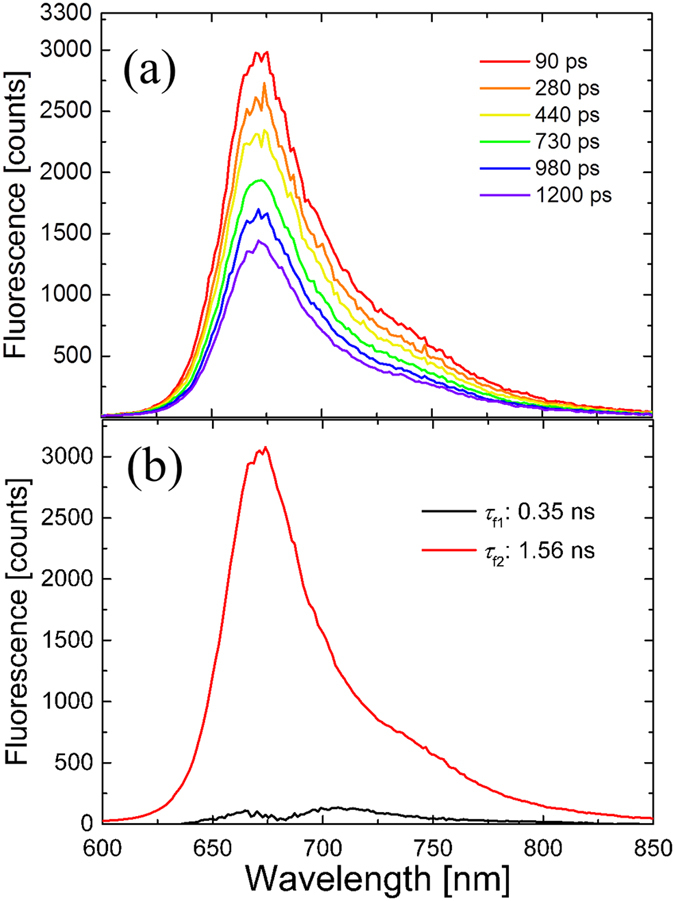
Picosecond time-resolved fluorescence spectra of BphP1-FP. (**a**) Selected time-resolved emission spectra and (**b**) decay-associated spectra (DAS) with 640 nm excitation.

**Figure 4:**
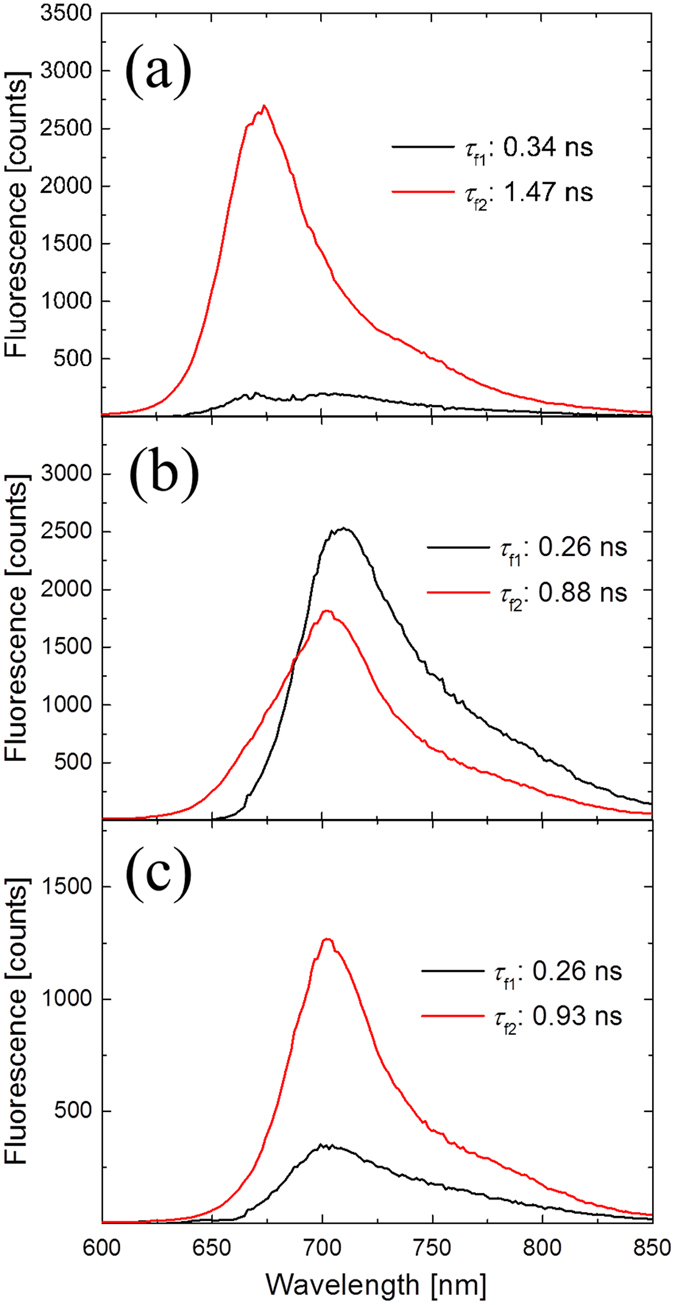
Decay-associated spectra (DAS) of picosecond time-resolved fluorescence in (**a**) BphP1-FP/C20S with 640 nm excitation, (**b**) BphP1-FP/C253I with 640 nm excitation and (**c**) BphP1-FP/C20S/C253I with 640 nm excitation.

**Figure 5:**
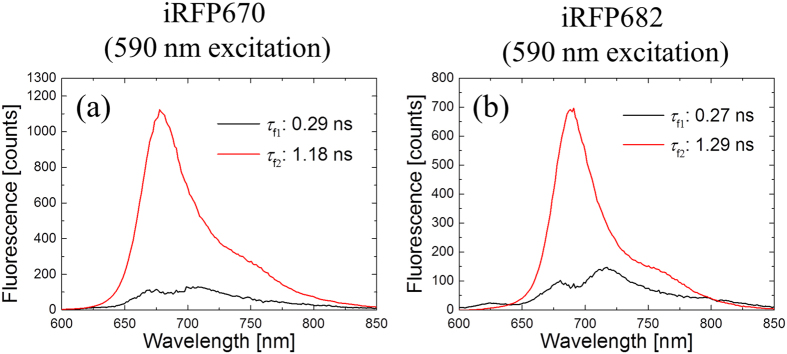
Decay-associated spectra (DAS) of picosecond time-resolved fluorescence in (**a**) iRFP670 and (**b**) iRFP682 with 590 nm excitation.

**Figure 6:**
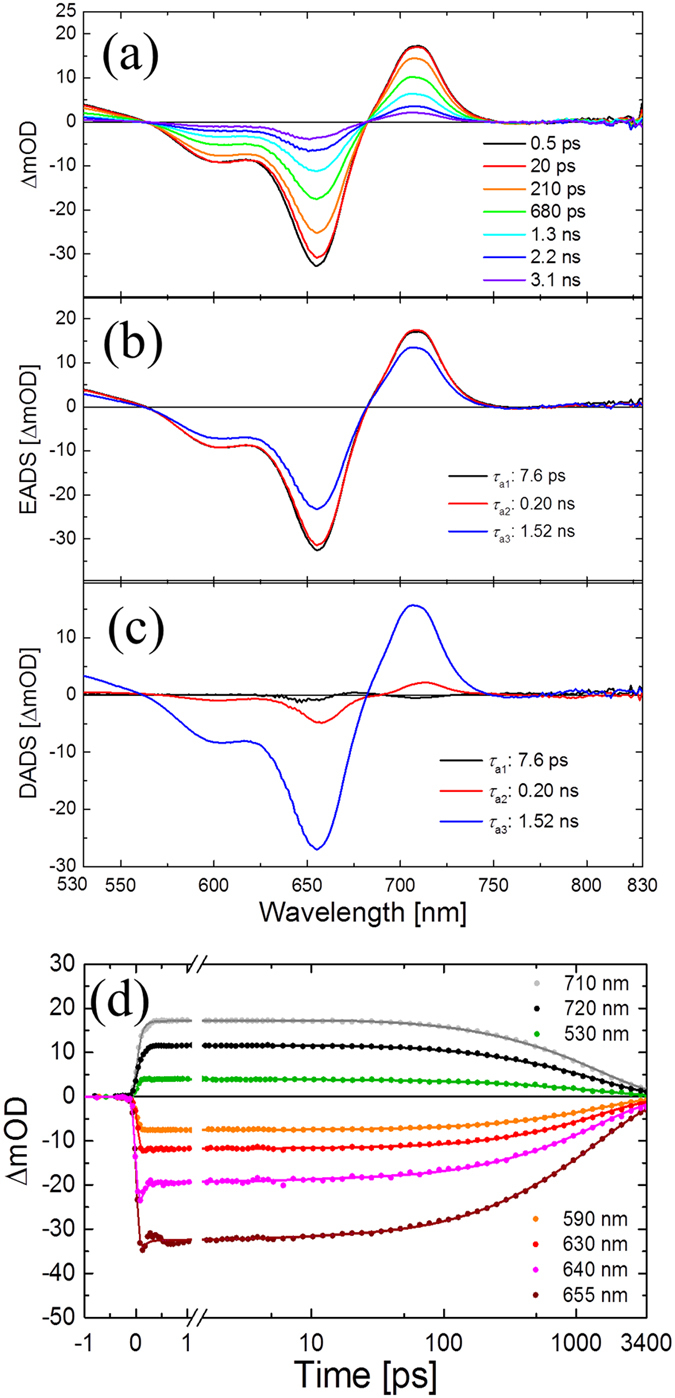
Femtosecond transient absorption spectra in BphP1-FP with 640 nm excitation. (**a**) Selected transient absorption spectra. (**b**) Evolution-associated difference spectra (EADS). (**c**) Decay-associated difference spectra (DADS). (**d**) Time traces of selected wavelengths.

**Figure 7:**
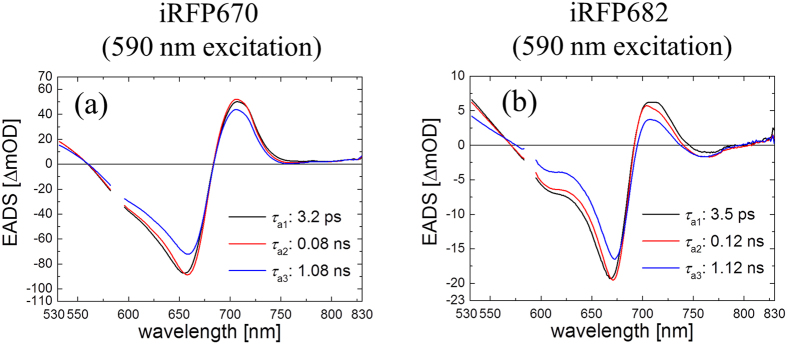
Evolution-associated difference spectra (EADS). Of femtosecond transient absorption in (**a**) iRFP670 and (**b**) iRFP682 with 590 nm excitation. Spectra region at 585–595 nm are cut because of the strong pump light scattering.

**Figure 8:**
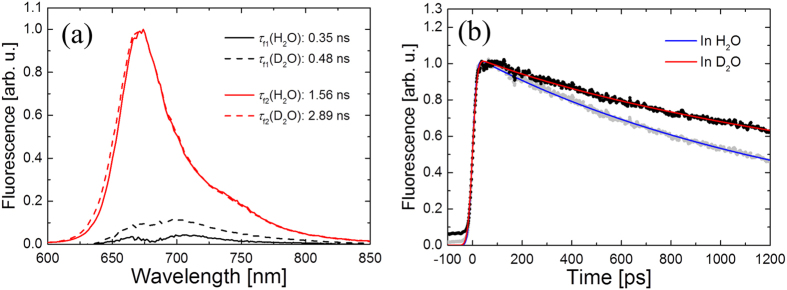
Time-resolved fluorescence spectra of BphP1-FP in H_2_O and D_2_O with 640 nm excitation. (**a**) Decay-associated spectra (DAS) in H_2_O (solid lines) and D_2_O (dashed lines). (**b**) Time traces of time-resolved emission at 670 nm in H_2_O and D_2_O with global fitted curves.

There should be two distinct blue-shifted emissions from BV binding to Cys253 via C3^1^ and C3^2^, whereas only one component is observed. Apparently, the two fluorescent species have emission spectra and lifetime too close to each other to be resolved in the time resolved measurements. This is likely the case because (i) the emission wavelength is mainly determined by the π-electron conjugation, which identical in both cases and to a lesser extent by pigment-protein interactions, which are highly similar for both cases, and (ii) the lifetime is mainly determined by pigment-protein interactions in the binding pocket to rings B, C and D, which is highly similar in both cases. We further note that in BphP1-FP and its C20S mutant, the steady-state absorption spectra (Fig. 2), transient fluorescence spectra (Figs 3b and 4a) and transient absorption spectra (Figs 6b and S5a) are almost identical. These spectra reflect structures of BV adducts in the protein, suggesting that BV in BphP1-FP forms almost the same adducts as in BphP1-FP/C20S. In other words, BphP1-FP has BV that forms a covalent bond with Cys253 in the GAF in two ways via C3^1^ and via C3^2^, but hardly binds Cys20 in the PAS domain. Possibly, the minor red-shifted species of BphP1-FP and its C20S mutant with weak 700-nm fluorescence originated from non-covalent BV-protein complex, like in the C20S/C253I mutant. Similar conclusions apply to iRFP670 and iRFP682 (Figs 7, S3 and S4).

Taken together, the static and time-resolved spectroscopic data on BphP1-FP and its mutants the 3D X-ray structural data^16^ and biochemical data^16^ indicate that the blue-shifted species of BphP1-FP are derived from the BV adducts covalently bound to Cys253 in the GAF domain via C3^1^ and via C3^2^.

### Fluorescence lifetime, quantum yield and protein design opportunities

The BphP1-FP fluorescence lifetime of 1.56 ns is by far the longest reported on BV-binding bacterial phytochromes^27^,^39^,^47^,^53^, and, in fact, is almost identical to that of allophycocyanin (APC)^54^,^55^, a cyanobacterial photosynthetic light harvesting complex that binds PCB in a ZZZasa configuration^56^. This notion is interesting because APC has been optimized through natural evolution for optimal resonant energy transfer, and the same molecular parameters need to be optimized for resonant energy transfer and fluorescence. The phycobilisome terminal emitter L_CM_, which transfers energy to the chlorophylls in the photosynthetic membrane, binds PCB in a ZZZssa configuration as in phytochromes^52^ and has a fluorescence lifetime of 1.2 ns^57^. The BphP1-FP fluorescence lifetime is almost as long as that of the intensely fluorescent Y176F mutant of cyanobacterial phytochrome Cph1 (1.8 ns)^10^,^58^, which binds PCB and is therefore not readily genetically encodable for *in vivo* fluorescence.

The blue-shifted fluorescence components of BphP1-FP, iRFP670 and iRFP682 have a higher quantum yield than red-shifted NIR FPs^11^,^14^. BphP1-FP has the highest quantum yield (~13%)^16^ and corresponding long fluorescence lifetime (1.56 ns) among the blue-shifted NIR FPs, which is twice higher than *e.g*. iRFP713 (~6.3%)^14^. The blue-shifted NIR FPs have a chromophore-binding Cys residue in the GAF domain, while the red-shifted NIR FPs have the chromophore-binding Cys only in the PAS domain, which clearly indicates that covalent binding of BV to the Cys in the GAF (via C3^1^ and C3^2^) is a key condition to achieve the high fluorescence quantum yield. Theoretical studies have indicated that ring A and ring D rotation followed by internal conversion constitute major causes of excited-state deactivation in bilin chromophores^59^, that will form an effective channel to deactivate fluorescence in NIR FPs and wild-type BphPs. Considering the above, two plausible factors may contribute to the higher fluorescence quantum yield of the proteins studied here: (i) the BV C3^1^/C3^2^ –Cys adduct in the GAF domain is sterically more constrained and leaves less mobility for ring A and by extension, the A-B methine bridge than in BphPs with the BV–Cys adduct in the PAS domain; (ii) differently from the red-shifted NIR FPs and wild-type BphPs, the π-electron conjugated system does not extend on the ring A in BphP1-FP (Fig. 1). Consequently, any ring A rotation will affect the π-electron conjugated system to a lesser extent than in traditional BphPs.

So far, most efforts to optimize the fluorescence quantum yield of BphP-based NIR FPs have been focused on restricting the motion of the BV D ring^13^,^14^,^60^. Here, we find that restricting ring A motion and/or locking out ring A from the conjugated system increases the fluorescence quantum yield. This result suggests that in general, restricting ring A mobility in red-shifted fluorescent BphP derivatives may prove beneficial for achieving higher brightness. This proposal seems to contradict an earlier study on BphPs with locked BV chromophores^61^, where restricting the motion of ring A did not have a signifcant effect on the fluorescence quantum yield. However, in those studies, the quantum yields were comparably low (<0.01), which implies that other pathways including ring D motion contributed to the fluorescence decay. It suggests that for a BphP with a low fluorescence quantum yield, ring D motion is limiting the fluorescence. We propose that as ring D is locked down by genetic engineering with a resulting higher fluorescence quantum yield, ring A motion becomes a limiting factor for the fluorescence quantum yield. In addition ESPT, which probably is in effect independently from ring D or A motion^47^, may limit the fluorescence quantum yield for longer fluorescence lifetimes^27^,^39^,^47^.

## Conclusions

We have systematically characterized the excited state dynamics and fluorescence deactivation mechanisms in the *Rp*BphP-derived blue-shifted NIR FPs: BphP1-FP, iRFP670, iRFP682, and their Cys mutants in the PAS and GAF domains. The substantially prolonged lifetimes and enhancement of fluorescence intensity were identified in the blue-shifted NIR FP variants, while the isomerization processes were found to be completely blocked. In the time-resolved fluorescence spectra, a distinct blue-shifted emission with a long lifetime was identified. Combining the results from ultrafast spectroscopy with the crystal structure of the BphP1-FP/C20S mutant and mutational analysis of the relevant Cys residues we assign the bright blue-shifted fluorescent species to two BV adducts, which are covalently bound to the Cys residue in the GAF domain. Our results explain the origin of high fluorescence quantum yield and blue-shifted spectra in NIR FPs with these BV adducts. Overall, these findings provide significant insights for the rational design of brighter and multicolor NIR FPs from a variety of wild-type BphPs.

## Additional Information

**How to cite this article**: Hontani, Y. *et al*. Bright blue-shifted fluorescent proteins with Cys in the GAF domain engineered from bacterial phytochromes: fluorescence mechanisms and excited-state dynamics. *Sci. Rep*. **6**, 37362; doi: 10.1038/srep37362 (2016).

**Publisher's note**: Springer Nature remains neutral with regard to jurisdictional claims in published maps and institutional affiliations.

## Supplementary Material

Supplementary Information
