# Supplementary material for: Bright blue-shifted fluorescent proteins with Cys in the GAF domain engineered from bacterial phytochromes: fluorescence mechanisms and excited-state dynamics
Source: Sci Rep. 2016 Nov 18;6:37362. doi: 10.1038/srep37362 (PMC5114657; doi:10.1038/srep37362)
Supplement: Supplementary Information [file srep37362-s1.pdf]

**Electronic Supplementary Information for:**

# Bright blue-shifted fluorescent proteins with Cys in GAF domain engineered from bacterial phytochromes: fluorescence mechanisms and excited-state dynamics

Yusaku Hontani[a], Daria M. Shcherbakova[b], Mikhail Baloban[b], Jingyi Zhu[a], Vladislav V. Verkhusha[b,c] and John T.M. Kennis[a]

[a] Department of Physics and Astronomy, VU University Amsterdam, Amsterdam 1081 HV, The Netherlands. [b] Departments of Anatomy and Structural Biology, Albert Einstein College of Medicine, Bronx, New York 10461, USA. [c] Department of Biochemistry and Developmental Biology, Faculty of Medicine, University of Helsinki, Helsinki 00290, Finland.

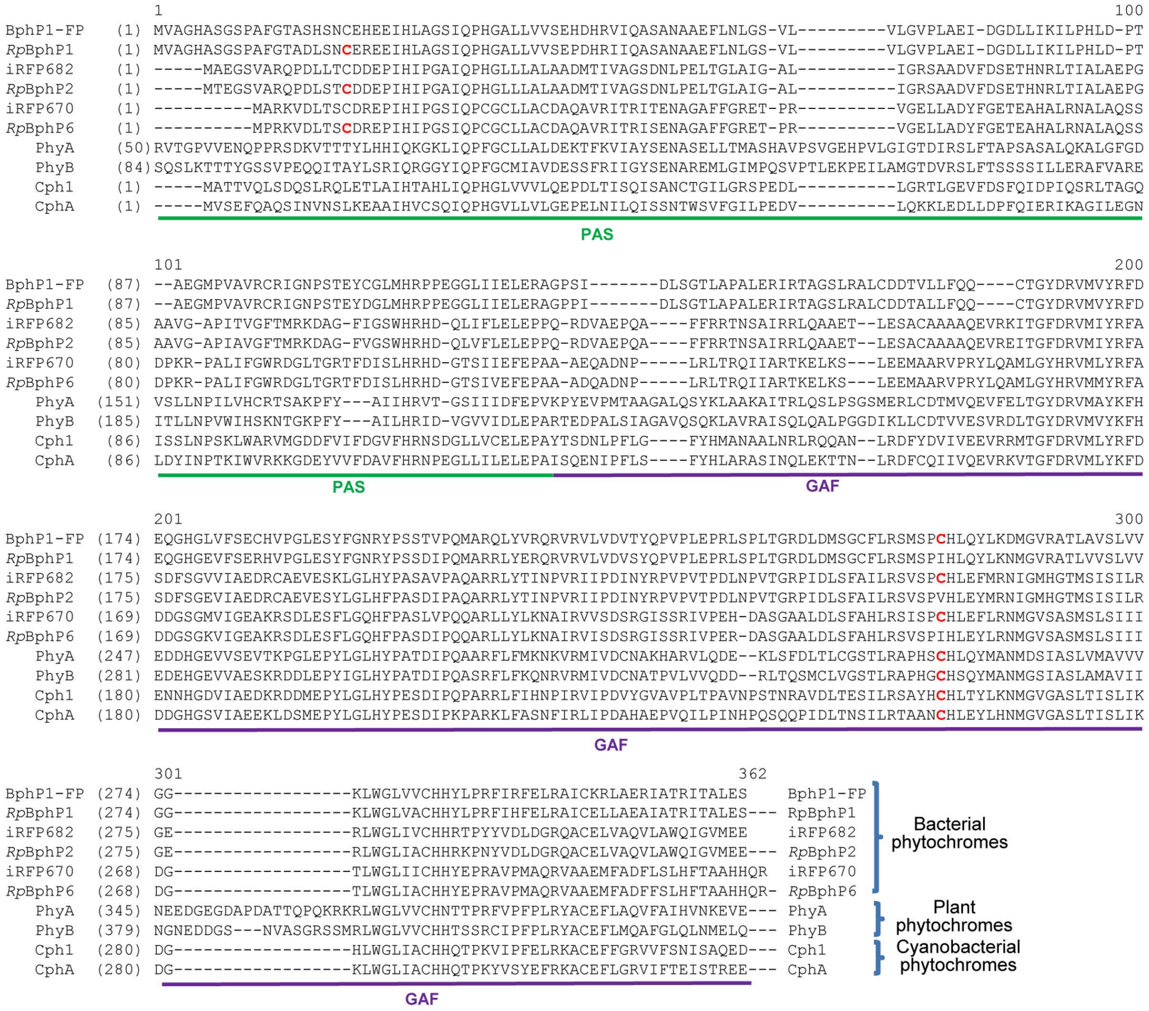


Fig. S1. Alignment of amino acid sequences of BphP1-FP, iRFP670 and iRFP682, which are blue-shifted NIR FPs with the PAS-GAF domains of their parental bacterial phytochromes (*Rp*BphP1, *Rp*BphP2 and *Rp*BphP6, respectively), as well as with selected plant phytochromes (PhyA and PhyB) and cyanobacterial phytochromes (Cph1 and CphA). The proposed chromophore binding Cys residues in the PAS and GAF domains are shown in red.

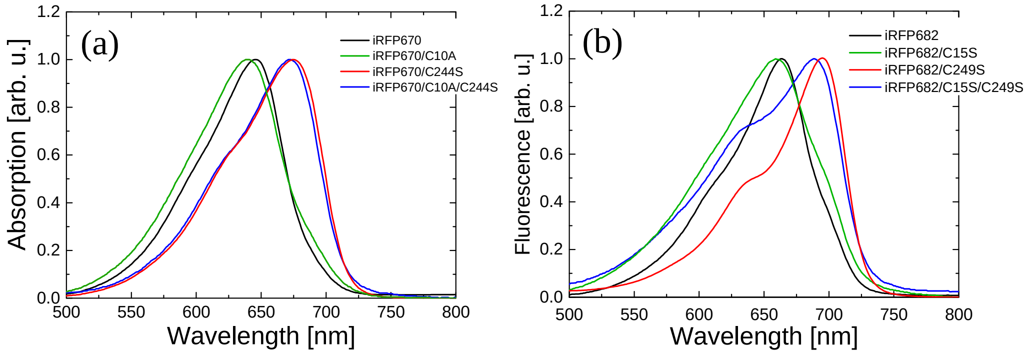


Fig. S2. Steady-state absorption spectra of (a) iRFP670 and its C10A, C244S and C10A/C244S mutants, and (b) iRFP682 and its C15S, C249S and C15S/C249S mutants.

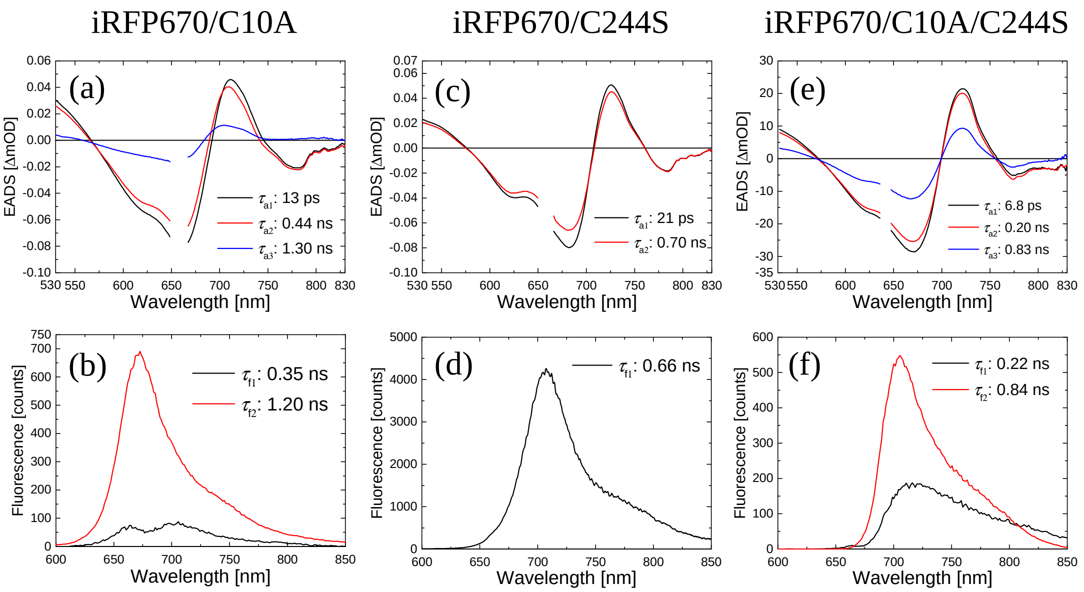


Fig. S3. EADS of transient absorption and DAS of transient fluorescence in (a, b) iRFP670/C10A (excitation at 660 nm and 590 nm, respectively), (c, d) iRFP670/C244S (excitation at 660 nm) and (e, f) iRFP670/C10A/C244S (excitation at 640 nm and 630 nm, respectively).

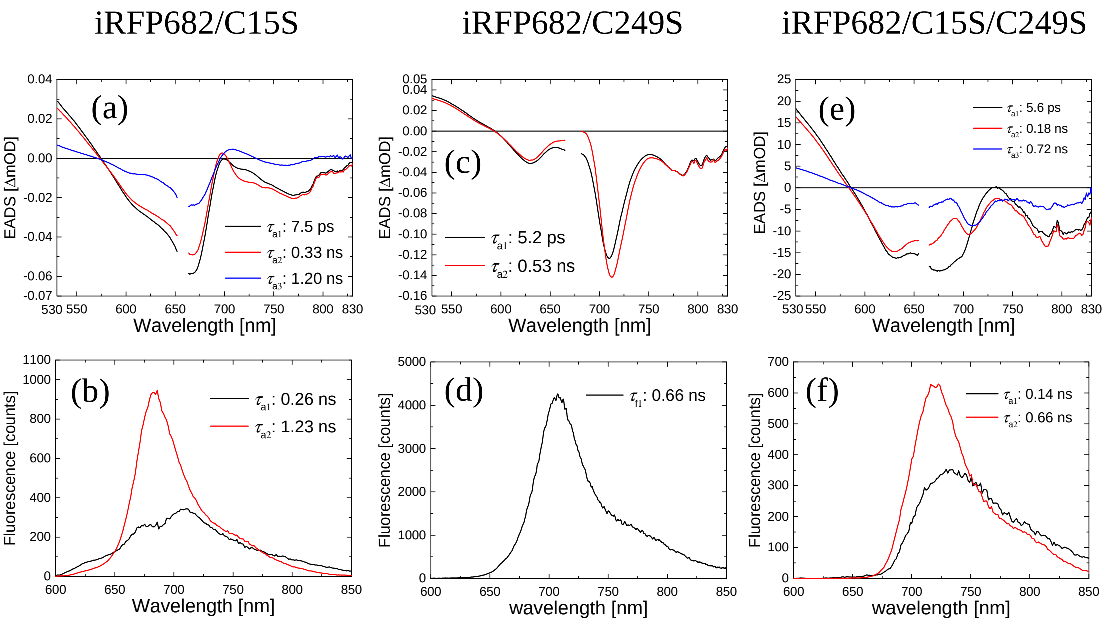


Fig. S4. EADS of transient absorption and DAS of transient fluorescence in (a, b) iRFP682/C15S (excitation at 660 nm and 590 nm, respectively), (c, d) iRFP682/C249S (excitation at 675 nm and 660 nm) and (e, f) iRFP682/C15S/C249S (excitation at 660 nm and 630 nm).

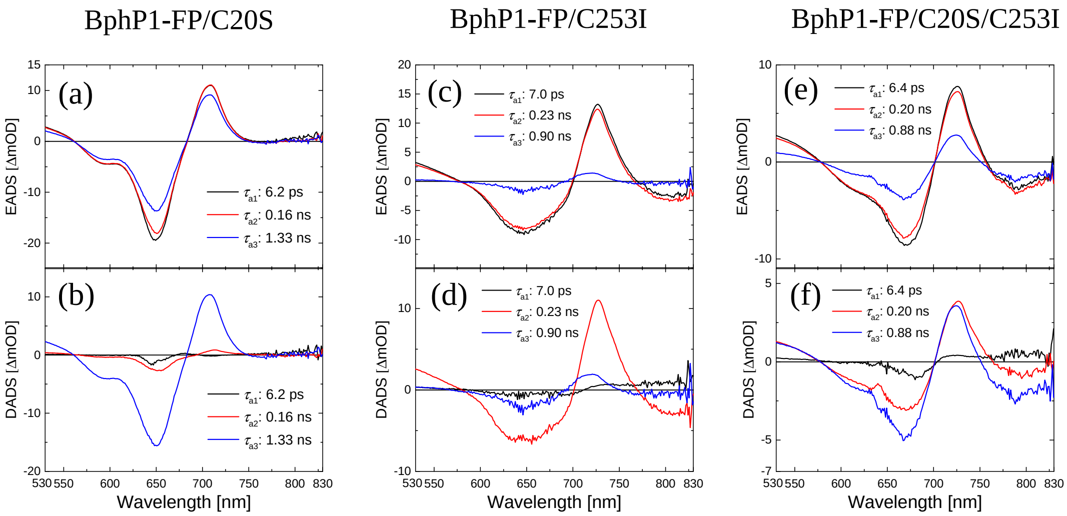


Fig. S5. EADS and DADS of transient absorption in (a, b) BphP1-FP/C20S, (c, d) BphP1-FP/C253I and (e, f) BphP1-FP/C20S/C253I with excitation at 640 nm.

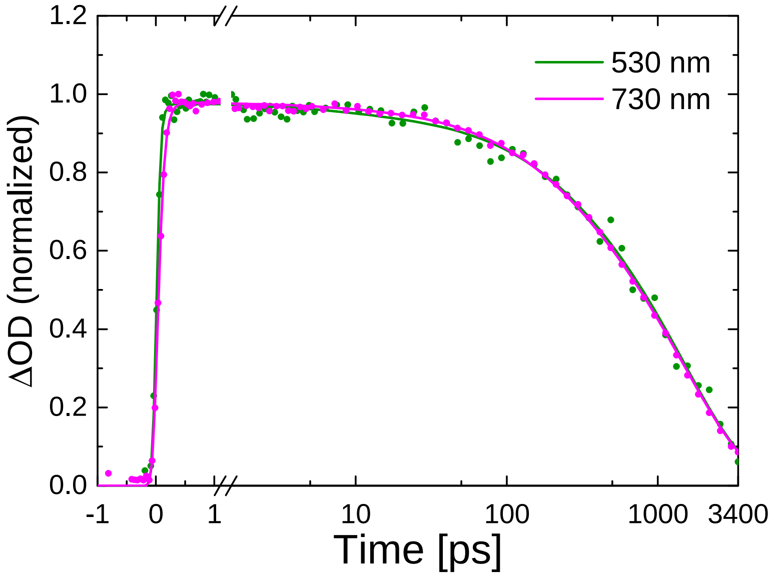


Fig. S6. Normalized time traces of transient absorption spectroscopy for BphP1-FP in $H_2O$ at 530 nm and 730 nm. The green and magenta closed-dots show the raw data at 530 nm and 730 nm, and the green and magenta lines show the global fitting results at 530 and 730 nm, respectively.

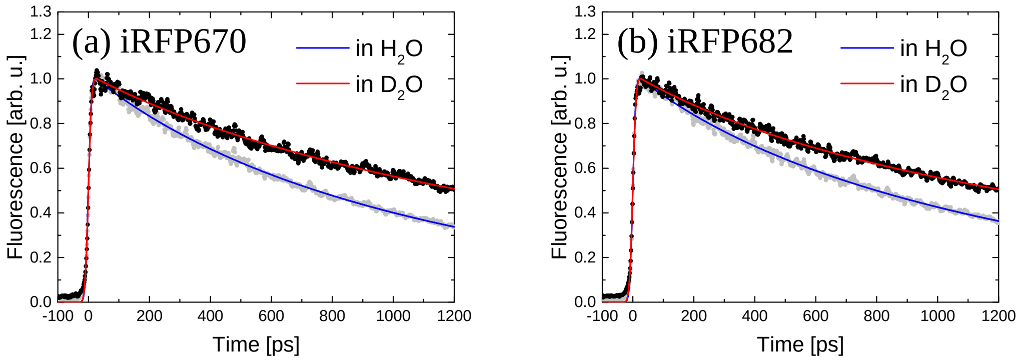


Fig. S7. Time traces of transient fluorescence of (a) iRFP670 in $H_2O$ and $D_2O$ at 675 nm with 590 nm excitation and (b) iRFP682 in $H_2O$ and $D_2O$ at 690 nm with 590 nm excitation.

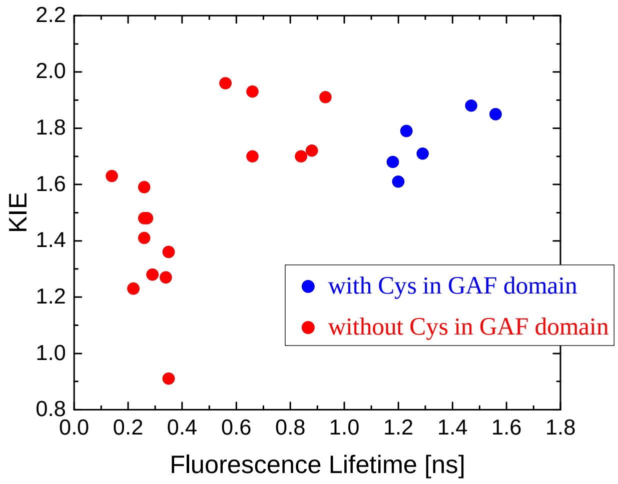


Fig. S8. H/D kinetic isotope effects (KIEs) of BphP1-FP, iRFP670, iRFP682 and their Cys mutants. Blue dots show KIEs of NIR FPs that have Cys in the GAF domain, and red dots show KIEs of NIR FPs without Cys in the GAF domain.

Table S1. Time constants of transient absorption and fluorescent spectra of BphP1-FP and its cysteine mutants. Populations and H/D kinetic isotope effect (KIE) of each decay are shown in the brackets.

| | Time-resolved fluorescence | | | Time-resolved absorption | | |
|---|---|---|---|---|---|---|
| | Excitation wavelength | $\tau_{f1}$ | $\tau_{f2}$ | Excitation wavelength | $\tau_{a2}$ | $\tau_{a3}$ |
| BphP1-FP | 640 nm | 0.35 ns (5%) **(KIE: 1.4)** | 1.56 ns (95%) **(1.9)** | 640 nm | 0.20 ns (amp. of GSB: -4.7) **(KIE: 1.2)** | 1.52 ns (-27.0) **(1.5)** |
| BphP1-FP/ C20S | 640 nm | 0.34 ns (10%) **(1.3)** | 1.47 ns (90%) **(1.9)** | 640 nm | 0.16 ns (-2.7) **(1.9)** | 1.33 ns (-15.5) **(1.7)** |
| BphP1-FP/ C253I | 640 nm | 0.26 ns (59%) **(1.5)** | 0.88 ns (41%) **(1.7)** | 640 nm | 0.23 ns (-6.0) **(1.7)** | 0.90 ns (-2.3) **(1.7)** |
| BphP1-FP/ C20S/C253I | 640 nm | 0.26 ns (29%) **(1.4)** | 0.93 ns (71%) **(1.9)** | 640 nm | 0.20 ns (-3.1) **(1.5)** | 0.88 ns (-4.8) **(1.6)** |
